# To: Association between rectus femoris cross-sectional area and diaphragmatic excursion with weaning of tracheostomized patients in the intensive care unit

**DOI:** 10.62675/2965-2774.20240131-en

**Published:** 2024-05-07

**Authors:** Josef Finsterer, Carla Alexandra Scorza, Antonio-Carlos Guimarães Almeida, Fulvio Alexandre Scorza

**Affiliations:** 1 Neurology & Neurophysiology Center Vienna Austria Neurology & Neurophysiology Center - Vienna, Austria.; 2 Universidade Federal de São Paulo Escola Paulista de Medicina Disciplina de Neurociência São Paulo SP Brasil Disciplina de Neurociência, Escola Paulista de Medicina, Universidade Federal de São Paulo - São Paulo (SP), Brasil.; 3 Universidade Federal de São Paulo Escola Paulista de Medicina Centro de Neurociências e Saúde da Mulher "Professor Geraldo Rodrigues de Lima" São Paulo SP Brasil Centro de Neurociências e Saúde da Mulher "Professor Geraldo Rodrigues de Lima", Escola Paulista de Medicina, Universidade Federal de São Paulo - São Paulo (SP), Brasil.

## To the Editor

We read an interesting prospective, single-center, observational cohort study on the relationship between the cross-sectional diameter of the rectus femoris muscle, the degree of diaphragmatic excursion, and the outcome of weaning 81 critically ill patients by Vieira et al.^(1)^ Successfully weaning critically ill patients from mechanical ventilation has been found to be associated with a larger cross-sectional area of the rectus femoris and diaphragmatic excursion.^(1)^ The study is compelling but has limitations that should be discussed.

The first limitation of the study is that the cross-sectional area of the rectus femoris muscle depends on several nonstandardized factors. The ultrasound measurement of the cross-sectional area of the rectus femoris depends on age, sex, caloric intake, diet, local arterial perfusion, physical condition of the patient before admission to the intensive care unit, innervation of the muscle, previous illness, comorbidities, and current medication. Therefore, few homogeneous cohorts can be generated, which makes the results unreliable.

A second limitation of the study is that diaphragmatic deflection can also depend on multiple factors, such as previous lung or bronchial diseases, diseases of the central nervous system or the peripheral nervous system (PNS), status of the neuromuscular junction, premorbid physical activity (training condition), muscle function, and current medications.

A third limitation of the study is the design. The design was that of a single-center study, which limits the generalizability of the data. Prospective, multicenter studies on homogenous cohorts are needed to answer the question of whether weaning success depends only on muscle trophics and the extent of diaphragmatic excursion.

Successful weaning depends not only on muscle volume and the extent of diaphragmatic excursions but also on various other conditions. These include exposure to infectious agents, immunological status, the presence or absence of central nervous system disease, the presence or absence of peripheral nervous system disease involving the phrenic nerve, the presence or absence of pulmonary and bronchial disease, the presence or absence of cardiac disease, nutritional factors, and metabolic factors. Muscle mass and contractility are several factors that are responsible for the success or failure of weaning. The success of weaning may also depend on the type and duration of mechanical ventilation and on the type and duration of complications occurring during mechanical ventilation, such as sepsis, pulmonary embolism, pneumothorax, emphysema, chronic pulmonary disease, heart failure, and coronary heart disease. We should therefore know, for example, how many of the included patients developed critical neuropathy or critical myopathy during their stay in the intensive care unit.

Overall, the limitations of this interesting study put the results and their interpretation into perspective. Addressing these issues would strengthen the conclusions and could improve the status of the study. Based on the considerations presented above, the outcome of weaning depends not only on the trophism of the nonrespiratory muscles and the contractility of the diaphragm but also on several other influencing factors.
