# Supplementary material for: To: Association between rectus femoris cross-sectional area and diaphragmatic excursion with weaning of tracheostomized patients in the intensive care unit
Source: Crit Care Sci. 2024 May 7;36:e20240131en. doi: 10.62675/2965-2774.20240131-en (PMC11098062; doi:10.62675/2965-2774.20240131-en)
Supplement: Translation

# Translation: Para: Associação entre área de secção transversa do reto femoral e excursão diafragmática com o desmame de pacientes com traqueostomia na unidade de terapia intensiva

**DOI:** 10.62675/2965-2774.20240131-pt

**Authors:** Josef Finsterer, Carla Alexandra Scorza, Antonio-Carlos Guimarães Almeida, Fulvio Alexandre Scorza

**Affiliations:** 1 Vienna Austria Neurology & Neurophysiology Center - Vienna, Austria.; 5 São Paulo SP Brasil Disciplina de Neurociência, Escola Paulista de Medicina, Universidade Federal de São Paulo - São Paulo (SP), Brasil.; 6 São Paulo SP Brasil Centro de Neurociências e Saúde da Mulher "Professor Geraldo Rodrigues de Lima", Escola Paulista de Medicina, Universidade Federal de São Paulo - São Paulo (SP), Brasil.

## Ao Editor

Lemos com interesse o estudo de coorte observacional, prospectivo, realizado em um único centro, sobre a relação entre o diâmetro da secção transversal do reto femoral, o grau de excursão diafragmática e o desfecho do desmame de 81 pacientes graves, realizado por Vieira et al.^(1)^ Foi constatado que o sucesso do desmame da ventilação mecânica em pacientes graves está associado a uma maior área da secção transversal do reto femoral e à excursão diafragmática.^(1)^ O estudo é convincente, mas tem limitações que devem ser discutidas.

A primeira limitação do estudo é que a área da secção transversal do reto femoral depende de vários fatores não padronizados. A medição por ultrassom da área da secção transversal do reto femoral depende da idade, sexo, ingestão calórica, dieta, perfusão arterial local, condição física do paciente antes da admissão na unidade de terapia intensiva, inervação do músculo, doença prévia, comorbidades e medicação em uso. Portanto, poucas coortes homogêneas podem ser geradas, o que torna os resultados pouco confiáveis.

Uma segunda limitação do estudo é que a deflexão diafragmática também pode depender de vários fatores, como doenças pulmonares ou brônquicas anteriores, doenças do sistema nervoso central ou do sistema nervoso periférico (SNP), condição da junção neuromuscular, atividade física pré-mórbida (condição de treinamento), função muscular e medicamentos em uso.

Uma terceira limitação do estudo é o desenho. Trata-se de um estudo em um único centro, o que limita a generalização dos dados. São necessários estudos prospectivos e multicêntricos em coortes homogêneas para responder à questão de se o sucesso do desmame depende apenas dos tróficos musculares e da extensão da excursão diafragmática.

O sucesso do desmame depende não apenas do volume muscular e da extensão das excursões diafragmáticas, mas também de várias outras condições. Entre elas estão a exposição a agentes infecciosos, o estado imunológico, a presença ou ausência de doença do sistema nervoso central, a presença ou ausência de doença do sistema nervoso periférico envolvendo o nervo frênico, a presença ou ausência de doença pulmonar e brônquica, a presença ou ausência de doença cardíaca, fatores nutricionais e fatores metabólicos. A massa muscular e a contratilidade são vários fatores responsáveis pelo sucesso ou fracasso do desmame. O sucesso do desmame também pode depender do tipo e da duração da ventilação mecânica e do tipo e da duração das complicações que ocorrem durante a ventilação mecânica, como sepse, embolia pulmonar, pneumotórax, enfisema, doença pulmonar crônica, insuficiência cardíaca e doença coronariana. Portanto, precisaríamos saber, por exemplo, quantos dos pacientes incluídos desenvolveram neuropatia grave ou miopatia grave durante sua permanência na unidade de terapia intensiva.

Em geral, as limitações desse interessante estudo colocam os resultados e sua interpretação em perspectiva. A abordagem dessas questões fortaleceria as conclusões e poderia melhorar o andamento do estudo. Com base nas considerações apresentadas acima, o desfecho do desmame depende não apenas do trofismo dos músculos não respiratórios e da contratilidade do diafragma, mas também de vários outros fatores influenciadores.
